# Target product profile and discovery and development path for novel cryptococcal disease treatments

**DOI:** 10.1016/j.jinf.2025.106643

**Published:** 2025-10-30

**Authors:** David B. Meya, Manu De Rycker, Ian H. Gilbert, William Hope, Justine Jelagat Odionyi, Michael Keegan, Angela Loyse, Pablo Moral-Lopez, Peter R. Williamson, Lionel K. Tan, Isabela Ribeiro, Timothy J. Miles

**Affiliations:** a Department of Internal Medicine, College of Health Sciences, Makerere University, Kampala, Uganda; b University of Dundee, Dundee, UK; c University of Liverpool, Antimicrobial Pharmacodynamics and Therapeutics, Liverpool, UK; d Drugs for Neglected Diseases Initiative (DNDi), Geneva, Switzerland; e ViiV Healthcare Ltd., London, UK; f City St George’s University of London, London, UK; g GSK, Tres Cantos, Spain; h Laboratory of Clinical Immunology and Microbiology (LCIM), National Institute of Allergy and Infectious Diseases (NIAID), National Institutes of Health (NIH), Bethesda, MD, USA

**Keywords:** Cryptococcal meningitis, *Cryptococcus neoformans*, Drug development, Human immunodeficiency virus, Low- and middle-income countries

## Abstract

*Cryptococcus neoformans* and *Cryptococcus gattii* are World Health Organization critical and medium priority pathogens, respectively. These mainly impact people with human immunodeficiency virus residing in low- and middle-income countries, but other patient groups and settings are also affected. The high global morbidity and mortality and the limitations of current treatments provided an impetus for the development of a target product profile (TPP) for new anti-cryptococcal agents. Key attributes of the TPP include improved safety, superior (or at least comparable) activity to current treatments against all syndromes across the full disease spectrum (cryptococcal meningitis, cryptococcal pneumonia, etc.), relevance for *C. neoformans* and *C. gattii*, suitability for all age groups, oral and intravenous formulations, an acceptable treatment regimen, minimal/manageable drug-drug interactions, thermostability, and a barrier to resistance at least as high as current options. The aim of this TPP, along with the suggested discovery and development paths, is to assist all stakeholders in the development of novel cryptococcal disease treatments.

## Introduction

*Cryptococcus* spp. are encapsulated yeasts that are typically found in decaying organic matter and soil, particularly that containing bird and animal droppings.^1^ Cryptococcal infection typically results from the inhalation of fungal spores or desiccated small yeasts.^2^ During initial pulmonary infection, clinical manifestations range from asymptomatic to pneumonia to acute respiratory distress syndrome.^3^ *Cryptococcus* spp. can survive and replicate within macrophages, with subsequent haematogenous dissemination throughout the host.

*Cryptococcus* spp. are neurotropic, and invasion of the central nervous system (CNS) results in cryptococcal meningitis (CM) and encephalitis. The term “cryptococcal meningoencephalitis” is an all-encompassing term that reflects the fact that multiple CNS sub-compartments are involved.^4^ Clinical manifestations of CM are variable and include fever, headache, lethargy, seizures, altered mental status, memory loss, raised intracranial pressure, and in severe cases, coma.^5,6^ CM has been associated with an all-cause mortality rate of approximately 25–55% at 10 weeks in various studies,^7–9^ depending on the treatment regimen and clinical context. However, prognosis (morbidity and mortality) among apparently immunocompetent people without classical risk factors tends to be poorer due to delayed diagnosis and frequent development of post-infectious inflammatory response syndromes.^5,10,11^

*Cryptococcus neoformans* is responsible for the vast majority of cryptococcal disease globally.^3^ It currently tops the World Health Organization (WHO) fungal priority pathogens list based on mortality, incidence, global distribution, 10-year trends, inpatient care, complications and sequelae, antifungal resistance, preventability, access to diagnostic tests, and evidence-based treatments.^12^ *Cryptococcus gattii* is less common, but has been associated with cryptococcal disease in apparently immunocompetent individuals^13^ as well as autoantibodies to the cytokine granulocyte-macrophage colony stimulating factor in 76% of immunocompetent patients.^14^ *C. gattii* is medium priority in the WHO fungal priority pathogens list.^12^

Herein, we discuss the global burden of cryptococcal disease, the limitations of currently available treatments, the development of a target product profile (TPP) for much-needed novel cryptococcal disease treatments, the minimal and preferred attributes of these novel treatments, and potential drug discovery and development pathways.

### Global burden

Cryptococcal disease most often occurs in people with impaired cellular immunity, especially in people with human immunodeficiency virus (HIV) who have advanced HIV disease (defined as cluster of differentiation 4 (CD4) count < 200 cells/mm^3^)^15^ or present with acquired immunodeficiency syndrome (AIDS).^3,5,16^ Others at increased risk of cryptococcal disease include solid organ transplant recipients and people with cancer, diabetes mellitus, autoimmune diseases, and those on long-term corticosteroids.^5,16,17^ However, some people with cryptococcal disease have no identifiable predisposing conditions.^16,17^

Before the HIV epidemic in the 1980s, cryptococcal disease was rare, but cases then increased rapidly.^6^ Following the introduction of antiretroviral therapy (ART) in the mid-1990s, there was a decline in cryptococcal disease in high-income countries.^16^ However, in low- and middle-income countries (LMICs), cryptococcal disease continues to be a considerable problem despite ART roll-out due to ART non-adherence and failure.^15^

A review^18^ of three studies that estimated the global burden of CM among people with HIV in 2007,^19^ 2014,^20^ and 2020^15^ highlighted a downward trend in estimated CM cases and cryptococcal deaths, likely due to improvements and expanded access to ART.^15,18–20^ However, CM was still estimated to account for 19% of AIDS-related deaths in 2020, with more than 80% of CM cases and deaths occurring in Africa and Asia & the Pacific.^15,18^ In addition to the estimated 152,000 CM cases among people with HIV in 2020, there were also an estimated 41,000 CM cases among people without HIV (approximately half of which were among those with no known underlying disease), for a total global estimate of over 190,000 cases that year.^21^

### Current CM treatment options and their limitations

The WHO currently recommends three phases for the treatment of CM in people with HIV: induction, consolidation, and maintenance.^22^ Based on the AMBIsome Therapy Induction OptimisatioN (AMBITION) trial,^7^ the preferred induction regimen is one dose of liposomal amphotericin B (L-AmB) followed by 14 days of flucytosine and fluconazole (Table 1).^22^ Alternative induction regimens include amphotericin B deoxycholate (D-AmB) + flucytosine + fluconazole OR fluconazole + (flucytosine or L-AmB or D-AmB), depending on availability (Table 1).^22^ The WHO also recommends pre-emptive fluconazole for screened individuals who test positive for cryptococcal antigen (i.e. asymptomatic antigenaemia), and fluconazole prophylaxis for people with HIV with CD4 count < 100 cells/mm^3^ if screening is not available.^22^

D-AmB, which was discovered in 1955,^23^ is used to treat various invasive fungal infections.^24^ As D-AmB is associated with acute and chronic toxicity (e.g. nephrotoxicity, anaemia, and hypokalaemia), this needs to be prevented, monitored, and managed.^22^ This need can be reduced, though not removed, with the use of a formulation in which the amphotericin molecule is incorporated within the lipid bilayer of liposomes (L-AmB).^25^ L-AmB is preferred as it has an improved therapeutic index (i.e. better safety) and similar efficacy.^7,22,25^ Other lipid complex formulations are available, but are not currently recommended by the WHO due to a lack of clinical evidence and because the lipid formulations are not interchangeable.^22^ Flucytosine is a pyrimidine analogue that was identified in the 1950s and fluconazole is a triazole that was patented in the 1980s.^23^ Before administering flucytosine, electrolytes, haematologic status, and renal status should be determined, and flucytosine should be used with extreme caution in patients with impaired renal function (including dose adjustments and monitoring).^26^ Fluconazole has rarely been associated with serious hepatic toxicity, anaphylaxis, exfoliative skin disorders, and QT interval prolongation.^27^

One disadvantage of most of the current treatment regimens is the requirement for intravenous administration of AmB, which can be challenging in resource-limited and rural settings. Fluconazole + flucytosine for 14 days is the only oral regimen, but 10-week mortality with this combination was higher in the Advancing Cryptococcal Meningitis Treatment for Africa (ACTA) trial than for D-AmB + flucytosine for 7 days (35% vs 24%).^8^ Additionally, flucytosine availability may be limited in LMICs, and WHO guidelines do not recommend the use of high-dose fluconazole monotherapy due to limited supportive evidence.^22^

Another disadvantage is the need for patient monitoring. For D-AmB (or standard-dose L-AmB plus fluconazole), potassium and creatinine (every other day) and haemoglobin (every week) monitoring is recommended for the duration of therapy, as is pre-hydration and electrolyte replacement before D-AmB infusions,^22^ which can be challenging in some settings. Patients on a single high dose of L-AmB should have their potassium and creatinine levels monitored on days 1 and 3 and haemoglobin tested on day 1 (and day 7 if they are still in hospital).^22^ Patients on flucytosine are recommended to have their full blood counts monitored.^22^ Therefore, there remains a need for safer and more tolerable treatments.

After induction, consolidation is with fluconazole for 8 weeks followed by maintenance with lower-dose fluconazole until immune reconstitution (defined as CD4 count > 200 cells/mm^3^) and viral load suppression on ART (Table 1).^22,28^ Other triazoles (i.e. itraconazole, voriconazole, posaconazole, isavuconazole) are not recommended in the WHO guidelines^22^ due to their different bioavailability, variable cerebrospinal fluid (CSF) penetration, cost, drug interactions, and lack of robust trials.^3^ Routine use of adjunctive systemic corticosteroids is not recommended in the WHO guidelines,^22^ based on the CryptoDex trial.^29^

ART-naïve people with HIV and CM can develop cryptococcal immune reconstitution inflammatory syndrome (IRIS) after initiating ART.^30–32^ While immune recovery is essential for managing CM, some trials have shown superior outcomes with delayed versus early initiation of ART,^30–32^ although a recent trial does not.^33^ Currently, WHO guidelines recommend that ART should be delayed until 4–6 weeks after initiation of anti-cryptococcal therapy.^22^

The WHO guidelines only cover the treatment of CM in people with HIV, while other guidelines from around the world cover HIV-negative populations with CM, including transplant recipients and patients with cancer.^28,34–38^ Of note, some of these do include itraconazole, voriconazole, posaconazole, and isavuconazole, but generally only if fluconazole is unavailable or contraindicated.^28,36,38^

### Development of the TPP

While recommended treatment regimens for cryptococcal disease have evolved over the last decade, we continue to use drugs that were developed in the last century. Considering that 10-week mortality in the AMBITION trial was almost 25% among people with HIV and CM,^7^ development of new drugs that could further reduce mortality and improve efficacy, feasibility, access, safety, and drug-drug interactions is a priority. We therefore developed a TPP to establish key features and performance specifications of new cryptococcal disease treatments. A TPP is a prerequisite to ensure that new products are developed to meet pre-specified health-related goals.^39^

Our TPP was the result of an initial workshop involving industry and academic drug developers, clinicians, and non-governmental organisations, with further refinement following review by other key stakeholders.

### Initial drafting phase

The initial drafting phase included a review of the advantages and challenges of current treatment regimens, including efficacy, adverse event profiles, and routes of administration. We also reviewed the epidemiology of cryptococcal disease to understand patient populations, geographical locations, and the spectrum of disease. After consideration of whether different TPPs would be required for different populations (e.g. HIV, non-HIV immunocompromised, and apparently immunocompetent), it was suggested that one TPP could be used for all populations. However, we note that these may have different drug-drug interactions and comorbidity considerations. Initially, LKT (an infectious disease physician), MK (an HIV clinical scientist), PM-L (a drug discovery scientist), and TJM (an infectious disease drug discovery leader) drafted and refined the TPP.

### Initial consultative phase

The draft TPP was reviewed by experts from ViiV Healthcare, the University of Dundee, and the Drugs for Neglected Diseases initiative (DNDi). This included expertise on drug discovery and development, clinical management of HIV and opportunistic infections, and general infectious diseases in resource-limited settings. The draft TPP was adjusted to include “minimal” and “preferred” TPPs and the preference for one TPP for HIV and non-HIV populations was confirmed.

### Expert consultative phase and finalisation of the TPP

A virtual workshop was held (AL, DBM, IR, LT, MDR, MK, PM-L, TJM, WH) to review all aspects of the TPP. The panel included experts in antifungal pharmacodynamics (PD) and therapeutics, infectious diseases drug development, clinical, laboratory research, and management of cryptococcal disease. The objectives were to review the draft TPP and understand the strengths and areas for further development. Each area of the TPP was discussed and debated during the workshop, with off-line follow-up to review the outputs of the workshop and further develop and refine the TPP.

In October 2023, the TPP was presented to the End AIDS Action Group (www.endaidsaction.group), which includes a broad range of participants including world-leading specialists in antifungal development, clinical research, and management of cryptococcal disease. Their feedback was incorporated into the TPP. The group was subsequently expanded to include PRW, who has expertise in managing non-HIV immunocompromised patients.

After consideration of the input from all the experts and groups consulted, the TPP was further refined. However, we acknowledge that the proposed TPP may require revision in the future as new scientific evidence emerges and following user engagement activities.

### Proposed minimal and preferred TPPs

**Fig. 1.**
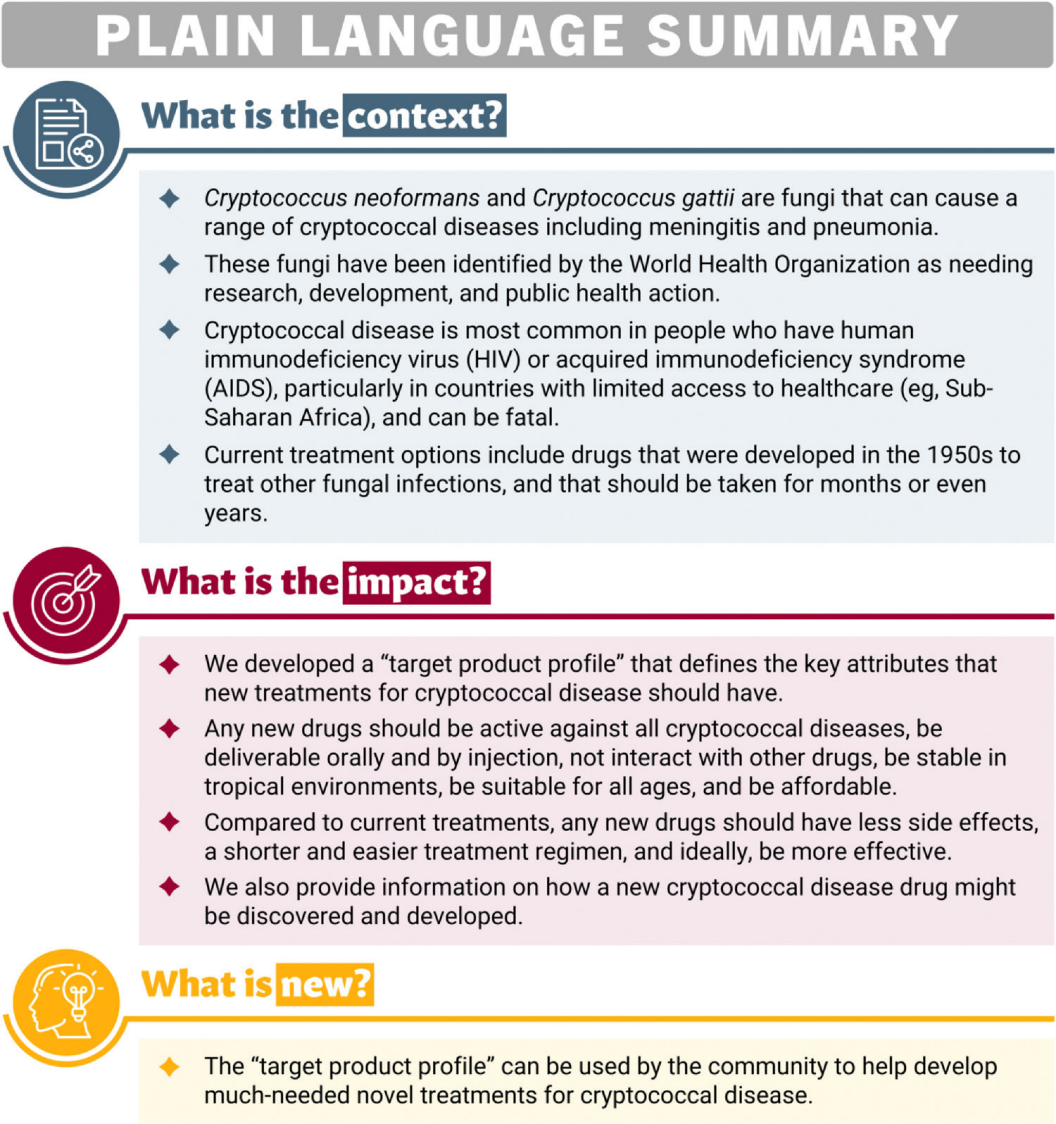
Plain language summary.

The proposed minimal and preferred TPPs are detailed below and summarised in Table 2, while Fig. 1 provides an overview in an easy-to-understand format that can be shared with end users.

### Indications for use

Any new treatment for cryptococcal disease, whether monotherapy or a combination of drugs, should be suitable for treating all disease severities, which can range from asymptomatic cryptococcal antigenaemia (i.e. cryptococcal antigen-positive [CrAg+]) to subclinical CM and overt/clinical CM.^40^

Any new treatment regimen should ideally be active against all cryptococcal species (particularly *C. neoformans*, but also *C. gattii*) and would be suitable for use as prophylaxis in individuals receiving a range of immunosuppressive modalities or regimens. As is common practice in anti-infective drug development, the inclusion of clinical strains of *C. neoformans* that are representative of different geographical areas early in the drug discovery process would be critical to ensure a robust therapeutic value of any novel antifungal. If any new cryptococcal drug were also active against other fungal species, this could be an additional benefit and increase the size of the population that would potentially use the drug.

### Target patient populations

A new drug or combination of drugs should be suitable for the treatment of immunocompromised and apparently immunocompetent adults (preferably also children). While there are some differences in the clinical presentation between immunocompromised and apparently immunocompetent individuals, there should be minimal differences in activity of the drugs depending on immune status. Ideally, new treatments would be suitable for use during pregnancy and lactation, which are not usually investigated early during drug development.

### Clinical efficacy

Any new treatment regimen would ideally be clinically and statistically superior to the gold standard of care, but, as this may be difficult to demonstrate, at a minimum it should have non-inferior efficacy and be superior in terms of other clinically relevant endpoints (e.g. tolerability and adverse event profile). A commonly used endpoint is 10-week mortality,^7,8^ while early fungicidal activity (i.e. rate of fungal clearance) is a potential surrogate endpoint that can reduce trial sample size, although this is still being validated.^41,42^ Currently, we recommend all-cause mortality as the primary endpoint, as it incorporates outcomes from drug efficacy as well as drug toxicity and drug interactions, but this could change if other endpoints are validated and approved by regulatory authorities.

Cryptococcal species are intrinsically resistant to the echinocandin class of antifungals and can also develop resistance to azoles and other antifungal drugs (as discussed below). It would be preferable if resistance were not a problem for any new cryptococcal disease treatment regimen, particularly considering the “One Health” approach focused on the interplay between human, animal, and environmental health and the use of antifungals within this ecosystem. It would, therefore, be useful to understand the resistance mechanisms and liabilities of any new medications.

### Safety/tolerability

Any new cryptococcal disease medication should have an improved safety and tolerability profile compared to the gold standard of care. Ideally, minimal clinical monitoring/supportive care would be required, as current therapy requires renal, electrolyte, hepatic, neutrophil, and haemoglobin monitoring, which is burdensome to both user and healthcare providers, particularly in resource-limited settings. As a preference, any new drugs should have good tolerability in children, who should not be precluded from novel treatments despite accounting for a small proportion of the disease burden.^43^ Ideally, any new drug would be non-teratogenic and be safe during breastfeeding to maximise its applicability.

### Formulation/presentation

Any new drug should ideally be easier to prepare and administer than current options. Given the high cost of intravenous administration (including the requirement for skilled workforce and monitoring) of drugs in hospital settings, oral agents are generally preferable for LMICs, with the option for nasogastric tube administration in those with CM and low levels of consciousness. Consideration should be given for alternative oral options for adults who cannot swallow tablets and children, e.g. oral or dispersible tablets or syrups, although these would need to be palatable. Other dosage forms should preferably be available for very severe disease (i.e. intravenous, intramuscular, or subcutaneous), although this may prove challenging for drug development, as different physicochemical properties are preferred for different dosage forms.

### Dose regimen

The current WHO-recommended treatment regimen for people with HIV is long (2-week induction, 8-week consolidation, and maintenance until immune reconstitution),^22^ as are regimens for other populations.^28,34–38^ Any new drugs should shorten this burdensome regimen. If possible, new treatments would have sufficiently long half-life, potency, and fungicidal activity so that a single agent could be administered once or twice daily for 2–4 weeks. Failing this, a twice-daily combination therapy with a 1-week induction, 4–6-week consolidation, and maintenance until immune reconstitution could be considered.

### Drug-drug interactions

Ideally, there would be few (or at least manageable) drug-drug interactions with common medications in appropriate populations, including ART for people with HIV, transplant-conditioning agents (e.g. sirolimus), antimalarials, antituberculosis agents, cotrimoxazole, and antifungals, considering the common comorbidities (e.g. HIV, cancer, diabetes). Additionally, due to the neurological localisation of CM, use of concomitant anticonvulsants may be necessary and hence a lack of interaction with all anticonvulsant therapies would be preferred. Lastly, although corticosteroids are not routinely recommended in the WHO guidelines for the treatment of CM in people with HIV,^22^ these may be necessary in other populations^35,44^ and patients on corticosteroids are at increased risk of CM,^5,17^ so any new treatments should not interact with these. Although minimal drug-drug interactions would make any new treatments suitable for a wider range of patients, we acknowledge that some interactions may preclude certain populations.

### Product stability and storage

The product should be heat stable, with a 3-year shelf life in hot/humid climates. A shelf-life of 3 years in hot/humid conditions is considered the minimum acceptable to allow distribution and administration of the product to the target patient population. If possible, it would have no requirement for refrigeration, considering the likely use in settings with limited resources to support cold chain maintenance.

### Product registration path

Although any new cryptococcal drugs will primarily be used in LMICs, by first intent, the data package from any clinical trials should support approval by a WHO Listed Authority.^45^ These can then be used to help with registration in countries with the highest burden of disease and WHO pre-qualification to enable access to the drug in these countries.

### Patient and payor value proposition

Overall, any new products should be novel, effective, safe, and suitable for the full spectrum of cryptococcal disease. It should not require hospitalisation for administration or monitoring and should not (or minimally) require monitoring for adverse events. Also, it should have no (or limited) contraindications, no (or minimal/manageable) interactions, and be suitable for combination use. Preferably, it should also have a reduced risk of inducing IRIS, have improved compliance (due to reduced toxicity, shorter duration, less frequent dosing, etc.), be CNS-penetrant (although this depends on blood–brain barrier [BBB] permeability as discussed below), and enable increased access to treatment. Although cryptococcal IRIS is due to an interplay of host immunological factors and pathogen factors, there is evidence to suggest that one of the factors associated with the risk of IRIS is fungal load pre-ART initiation.^46–48^ We thus hypothesise that if a cryptococcal sterilising cure could be successfully developed, or at least a treatment that significantly reduces the fungal burden rapidly, this has the potential to decrease the risk of cryptococcal IRIS, although this would need to be proven in clinical studies.

Cost is also an important factor, given that most cases occur in Africa.^15^ Preferably, it would be affordable and supported by robust cost-effectiveness analysis taking into consideration the cost of manufacture of the product (cost of goods) and also any cost savings to the health service due to improved efficacy and safety.

### Discovery and development path

Antifungal development is challenging because fungal pathogens have various biological processes in common with humans.^49^ Activity against fungal pathogens is therefore likely to result in toxicity to humans.^49^ Drug discovery efforts for CM are limited, with the furthest developed series being new formulations or repurposed from other antifungal drug discovery efforts. These include an oral lipid nanocrystal formulation of AmB (MAT2203),^50^ azoles (quilseconazole [VT-1129],^51^ oteseconazole [VT-1161],^52^ VT-1598,^53^ and opelconazole [PC945]^54^), glycosylphosphatidylinositol anchor biosynthesis inhibitors (fosmanogepix [APX001]^55^ and APX2039^55,56^), a diamidine (ATI-2307 [T-2307]),^57^ an acetyl coenzyme A ligase inhibitor (AR-12 [OSU-03012]),^58^ and a next-generation polyene drug (SF001).^59^ Limited earlier-stage efforts are also ongoing.^60–63^ Further details can be found in a recently published review of antifungals that are in development.^64^ Cryptococcus spp. are unique pathogens, evolutionarily divergent from most other human fungal pathogens such as *Aspergillus* spp. and *Candida* spp. In addition to the current repurposing efforts from other antifungal programmes, there is a need to develop fit-for-purpose *Cryptococcus*-specific drug discovery approaches.

### *In vitro* assays

Successful drug discovery relies on appropriate cell-based models that are predictive of clinical efficacy.^65^ In infectious diseases, this frequently requires testing compounds in multiple cell-based models that reflect the complexity of the pathogen and its interactions with the host.^66^ Despite being unicellular, *C. neoformans* displays remarkable heterogeneity in human infections,^3^ with various degrees of encapsulation, melanisation, and Titan cell formation as well as infection of macrophages and microglia. Assessing the value of preclinical compound screening assays that cover this heterogeneity is a key aspect of developing the critical path for the discovery and development of new cryptococcosis drugs, with the relevant assays potentially differing by indication (e.g. CM vs systemic disease). Capsule size, for example, could be a key factor in drug discovery as the capsule may limit compound permeability, which is particularly relevant as cryptococci isolated from the CSF of people with HIV show a range of capsule sizes, with capsule size associated with virulence.^67^ Backtranslation experiments, testing reference treatments, and new lead compounds in a range of preclinical cell-based models will be essential in this respect. Further triaging of hits should consider chemical tractability, potential to cross the BBB, and mode of action (avoiding compounds with the same mode of action as clinically used drugs).

### *In vivo* models

Laboratory animal models of CM are well characterised and highly predictive of therapeutic outcomes.^56,68,69^ These experimental tools have been pivotal in understanding the PD of antifungal agents including the emergence of resistance (e.g. to fluconazole),^70^ defining the optimal duration of induction therapies (e.g. D-AmB, L-AmB),^69,71^ defining the optimal combinations of antifungal agents,^53^ and defining the PD of new antifungal agents (e.g. APX2039 and ATI-2307).^56,72^ There are two model systems (murine and rabbits) that have strengths and limitations and yield complementary information.^69^ Data from both are often required to achieve a detailed understanding of dose-effect-response relationships. Both models offer the opportunity to describe the temporal change in fungal burden from unrestrained growth or drug-induced killing. Increased use of organoids has recently been proposed to minimise the use of animal models in drug development, including that stated in the Food and Drug Administration Modernization Act 2.0 in the U.S. However, more work will be needed to optimise these model systems for effective drug development generally and antifungal drug development specifically.^73^

#### Murine model

Mice are inherently susceptible to cryptococcal infection and do not require systemic immunosuppression to enable infection to be established. Intravenous inoculation results in disseminated infection with highly reproducible encephalitis. The endpoint for the estimation of the PD of antifungal agents is fungal burden in the brain.^69^ The mouse is too small to enable drug partitioning and PD in sub-compartments of the CNS. Rather, the cerebrum is considered a single and fully homogenous compartment.

Advantages of murine studies are that they are relatively straightforward to conduct, result in a highly reproducible encephalitis, and are highly predictive of clinical outcomes for different antifungal agents and classes.^74^ One key disadvantage is the inability to examine the CSF^69^ (which is the most relevant compartment for clinical disease and clinical studies). Uncertainty about effect site pharmacokinetics (PK) (i.e. concentrations in the cerebrum) means that PK–PD relationships are generally quantified in terms of plasma concentrations. This requires an assumption that tracking of drug from plasma to the site of infection is the same in mice as in humans. The use of PK to predict tissue concentrations can also be problematic, especially for lipid formulations that may have very different patterns of tissue partitioning.

#### Rabbit model

The rabbit model of CM requires intracisternal inoculation of *Cryptococcus*, which results in a reproducible meningoencephalitis.^72^ The model enables serial quantification of fungal burden in the CSF, which mimics clinical therapeutics. The rate of decline of fungal burden can be used for regimen identification and estimation of PD relationships.

Advantages of rabbit studies are that they closely mimic clinical disease and therapeutics, and effect site drug partitioning studies are relatively straightforward. Disadvantages are that they are resource intensive and significant technical expertise is required for intracisternal inoculation and serial CSF sampling, including advanced expertise in anaesthesia.

### Compound properties

Defining the appropriate compound properties for a new CM drug is important to guide drug discovery efforts but is not straightforward. Compounds need to penetrate the capsule and cell wall, a challenge exacerbated by their increased size and density in Titan cells.^75^ The physicochemical properties required to penetrate the *Cryptococcus* capsule and cell wall remain poorly understood. In addition, the complex pathophysiology of CM poses a challenge for establishing the required compound distribution properties. *Cryptococcus* is typically present in the meninges but can also be found in the brain parenchyma, usually in cryptococcomas.^76^ Increased BBB permeability in patients with CM^77^ allows non-brain penetrant compounds to reach the yeast cells and BBB permeability is thus not a prerequisite for efficacy. However, whether the BBB is always compromised, particularly in patients with subclinical CM, remains to be understood. As compounds progress, detailed CNS partitioning studies in both uninfected laboratory animal models and disease models are advised and should be considered in conjunction with pharmacodynamic data when deciding to advance compounds. From a safety perspective, it must be considered that for non-BBB penetrant compounds, standard preclinical toxicology and Phase 1 clinical trials may not identify CNS toxicities that could manifest in CM patients with a compromised BBB. Of note, early assessment of potential toxicity liabilities is paramount. Amphotericin B, developed in the 1950s, when regulatory requirements were less stringent, displays excellent efficacy, but its safety profile limits widespread use.^24^ In current drug discovery practice, safety is integrated right from the start, and new treatments are only developed if they combine efficacy with a suitable therapeutic index. Finally, compounds should ideally be orally available and stable in tropical zones.

### Clinical trials

The European Medicines Agency published guidelines on the clinical development of antifungals for treating invasive fungal disease in 2010.^78^ This includes guidance on the collection of preclinical data, including PK/PD and resistance considerations. Also, study design considerations for clinical trials, including disease categories, end users (including children), treatment types, biomarkers, and outcomes (including safety).^78^ Adaptive designs^79^ may be useful for assessing monotherapies, while combinations could be compared to one part of the combination or an approved monotherapy.^78^ Ideally, the comparator regimen for HIV-associated CM should consist of the preferred induction regimen recommended by WHO guidelines and the WHO-listed regulatory authorities at the time of the trial. For example, the WHO 2022 guidelines (see Table 1)^22^ are based on the results of the AMBITION study.^7^ This allows any medicine developed to be compared to the best available regimen.

Of note, there is a marked paucity of data on the use of antifungal CM regimens in non-HIV patients, especially in apparently immunocompetent individuals. The optimal duration of therapy at all stages (induction, consolidation, and maintenance) in this group is unclear and adequate evidence is necessary. Studies have been hampered by the lower number of cases^21^ and differences in treatment centres from those caring for people with HIV, leading to difficulty in conducting appropriately powered studies. Potential solutions could be to create patient post-approval registries or ongoing cohorts of patients such as the Cryptococcus Infection Network in non-HIV Cohort (CINCH),^80^ layering on adaptive designs that might explore multiple treatments, doses, durations, or combinations with options to ‘drop losers’ or ‘select winners’ early.^79^ Another challenge related to non-HIV patients is the frequency of post-infectious inflammatory sequelae that may require adjunctive immune modulation to optimise clinical efficacy.^44,81^ Treatment guidelines based on expert consensus have been published and the recommended regimens could serve as possible control regimens in studies.^28,34–37^

One way to decrease the required sample size of randomised controlled trials in CM could be to use early fungicidal activity as a surrogate endpoint for all-cause mortality.^42^ Such an approach may also be useful to select candidate interventions in phase 2 studies.^42^ In a study that investigated predictors of clinical outcome in people hospitalised with CM in a hospital in Brazil, higher CSF yeast count was found to predict in-hospital mortality and severity.^82^ However, such tests are invasive and have an associated cost, and given the acute nature of CM, all-cause mortality remains a suitable endpoint.^42^

### Resistance

While resistance to AmB is limited,^24^ in vitro resistance to flucytosine monotherapy emerges rapidly,^83^ and resistance to fluconazole is increasing.^84,85^ Human-to-human transmission of *Cryptococcus* is uncommon,^18^ but has been reported in transplant recipients.^86^ Most resistance is, therefore, either pre-existing in the natural population or emerges during patient treatment. Two resistance mechanisms are common in fungi, namely heritable and transient.^87^ Heritable resistance refers to stable point mutations or gene duplications, whereas transient resistance or heteroresistance is usually due to unstable aneuploidy. Of note, heteroresistance may be transient, but not all transient resistance is heteroresistance.

Resistance to flucytosine could result from mutations in the enzymes involved in flucytosine uptake and metabolism, or upregulation of pyrimidine synthesis.^83^ For fluconazole, both mutations in its target gene (*ERG11*) and heteroresistance have been observed.^84,88^ Heteroresistance is intrinsic in *Cryptococcus* (i.e. is found prior to treatment) and is driven by transient aneuploidy in a sub-population of cells.^70,89^ The generation of heteroresistance in *Cryptococcus* is facilitated by their unique Titan cells, which produce large numbers of aneuploid progeny.^87^ To overcome the resistance challenge, combination treatments can be used, as well as the development of drugs with novel modes of action.^90^

## Conclusions

*C. neoformans* was highlighted as critical in the 2022 WHO fungal priority pathogens list^12^ due to its public health importance and unmet research and development needs. As current drugs have important limitations, a new, affordable, oral therapy that enables safe and effective treatment in the community for cryptococcal disease could lead to increased access and the avoidance of costs associated with hospitalisation for parenteral dosing and the need for adverse event monitoring. Ideally, a new oral therapy would be adaptable for intravenous/intramuscular/subcutaneous use in the most severe cases and be suitable for treating immunocompromised and apparently immunocompetent individuals. A novel mode of action also opens up the possibility for its use as part of combination treatment to shorten treatment durations and minimise the potential for resistance. If possible, a new therapy should have superior safety and efficacy, a less cumbersome treatment regimen, and have no – or at least manageable – drug interactions.

Drug discovery efforts for CM are limited, with most being repurposed from other drugs. Given the unique nature of *Cryptococcus*, there is a need to develop targeted approaches.

This TPP provides a foundation for the development of a novel cryptococcal disease treatment to allow academic drug developers, pharmaceutical companies, and key stakeholders to understand the required characteristics. Given the advancement in the drug development arena, we believe we can be optimistic for the community to develop new drugs that can approach our idealistic TPP. As new scientific evidence is generated, together with input from patient engagement on the TPP for CM, this TPP may require further review and revision.

## Figures and Tables

**Table 1:** 2022 WHO guidelines for the treatment of CM in people with HIV.^22^

Preferred induction phase regimen
• One high dose of L-AmB (10 mg/kg) and
• 14 days of flucytosine (100 mg/kg/day^a^) and fluconazole (1200 mg/day for adults^b^)
Alternative induction phase regimens
If L-AmB is not available:
• 7 days of D-AmB (1 mg/kg/day) and flucytosine (100 mg/kg/day^a^) then
• 7 days of fluconazole (1200 mg/day for adults^b^)
If L-AmB and D-AmB are not available:
• 14 days of flucytosine (100 mg/kg/day^a^) and fluconazole (1200 mg/day for adults^b^)
If flucytosine is not available:
• 14 days of L-AmB (3–4 mg/kg/day) and fluconazole (1200 mg/day for adults^b^)
If L-AmB and flucytosine are not available:
• 14 days of D-AmB (1 mg/kg/day) and fluconazole (1200 mg/day for adults^b^)
Consolidation phase
• Fluconazole (800 mg/day for adults^c^) for 8 weeks following induction
Maintenance phase
• Fluconazole (200 mg/day for adults^d^) until immune reconstitution (CD4 > 200 cells/mm^3^) and suppression of viral loads on ART

Abbreviations: ART, antiretroviral therapy; CD4, cluster of differentiation 4; CM, cryptococcal meningitis; D-AmB, amphotericin B deoxycholate; HIV, human immunodeficiency virus; L-AmB, liposomal amphotericin B; WHO, World Health Organization.

a Divided into four doses per day.

b 12 mg/kg/day for children and adolescents up to a maximum of 800 mg/day.

c 6–12 mg/kg/day for children and adolescents up to a maximum of 800 mg/day.

d 6 mg/kg/day for adolescents and children.

**Table 2:** TPP for cryptococcal disease including CM.

Attribute	Minimal TPP	Preferred TPP

Indication(s) for use	• Tx of symptomatic and subclinical CM	• Tx of symptomatic and subclinical CM
	• Tx of symptomatic systemic cryptococcal disease (i.e. pulmonary, skin, lymph node, and bone)	• Tx of symptomatic systemic cryptococcal disease (i.e. pulmonary, skin, lymph node, and bone)
	• Tx of asymptomatic people who are CrAg+	• Tx of asymptomatic people who are CrAg+
	• Activity against all species	• Activity against all species
		• Prophylaxis against cryptococcal disease
Target patient populations	• Apparently immunocompetent and immunocompromised adults and adolescents with symptomatic CM	• Apparently immunocompetent and immunocompromised adults and children with CM
	• Apparently immunocompetent and immunocompromised adults and adolescents with symptomatic systemic cryptococcal disease (i.e. pulmonary, skin, lymph node, and bone)	• Apparently immunocompetent and immunocompromised adults and children with symptomatic systemic cryptococcal disease (i.e. pulmonary, skin, lymph node, and bone)
	• Apparently immunocompetent and immunocompromised CrAg+ adults and children	• Apparently immunocompetent and immunocompromised CrAg+ adults and children
		• Asymptomatic CrAg+ adults and children
		• Including use during pregnancy and lactation
Clinical efficacy	• Non-inferior to SoC^a^ with activity against all causative species	• Superior to SoC^a^ with activity against all causative species
		• High barrier to resistance
Safety/tolerability	• Improved safety and tolerability profile compared to current SoC^b^	• Improved safety and tolerability profile compared to current SoC^b^
		• Minimal or no requirement for monitoring (current therapy requires renal, hepatic, and anaemia monitoring)
		• Good tolerability in children
		• Non-teratogenic and improved safety during breastfeeding
Formulation/presentation	• Oral (+ nasogastric) and intravenous injection	• Easier administration
		• Oral (+ nasogastric) and intravenous, intramuscular, and subcutaneous injections
		• Good palatability and swallowability in adults with swallowing difficulties and children
Dose regimen	• Twice daily	• Once or twice daily
	• Part of combination therapy	• Single drug
	• Induction: 1 week; consolidation: 4–6 weeks; (maintenance: until immune reconstitution^c^)	• Total duration: 2–4 weeks (sterilising - no requirement for induction/consolidation/maintenance)
Drug-drug interactions	• Minimal/manageable drug interactions with concomitant medications (i.e. ART, transplant-conditioning agents, antimalarials, antituberculosis, cotrimoxazole, antifungals, anticonvulsants, corticosteroids, etc.)	• No drug interactions with concomitant medications (i.e. ART, antimalarials, antituberculosis, cotrimoxazole, antifungals, anticonvulsants, corticosteroids, etc.)
Product stability and storage	• Heat stable, 3-year shelf-life in hot tropic/humid climate (simulated with 30°C and 65% relative humidity)	• Heat stable, 3-year shelf-life in hot tropic/humid climate (simulated with 30°C and 65% relative humidity)
		• Stability of formulated suspension/intravenous formulations for multiple days without requirement for refrigeration
Product registration path	• WHO-listed authority approval followed by WHO prequalification	• WHO-listed authority approval followed by WHO prequalification
Patient and payor value proposition	• Novel, effective, and safe	• Novel, effective, and safe
	• Suitable for full spectrum of affected populations^d^	• Suitable for full spectrum of affected populations^d^
	• No requirement for hospitalisation specifically to administer or monitor treatment	• No requirement for hospitalisation specifically to administer or monitor treatment
	• Reduction in required monitoring for adverse events	• No adverse events requiring close monitoring
	• Contraindication: pregnancy/lactation	• No contraindications/interactions (suitable for use in combination)
	• Minimal/manageable drug interactions	• Reduction in incidence of IRIS
	• Potential for use as part of combination regimen	• Improved compliance due to shorter duration of therapy
	• CNS penetrant^e^	• CNS penetrant^e^
	• Increased access to treatment	• Increased access to treatment
	• Affordable target cost informed by cost-effectiveness analysis	• Affordable target cost informed by cost-effectiveness analysis

Abbreviations: ART, antiretroviral therapy; BBB, blood-brain barrier; CM, cryptococcal meningitis; CNS, central nervous system; CrAg+, cryptococcal antigen positive; CYP2C9, Cytochrome P450 family 2 subfamily C member 9; CYP3A4, Cytochrome P450 family 3 subfamily A member 4; D-AmB, amphotericin B deoxycholate; HIV, human immunodeficiency virus; IRIS, immune reconstitution inflammatory syndrome; L-AmB, liposomal amphotericin B; QT, length of time between the start of the Q-wave and the end of the T-wave; SoC, standard of care; TPP, target product profile; Tx, treatment; WHO, World Health Organization.

a L-AmB + flucytosine + fluconazole followed by fluconazole for people with HIV (see Table 1) or other gold standard treatment in other populations.

b D-AmB/L-AmB - anaphylaxis, gastrointestinal adverse events, rigors, fever, hypertension or hypotension, hypoxia, nephrotoxicity; flucytosine - gastrointestinal adverse events, hepatic adverse events, bone-marrow suppression, teratogenicity; fluconazole - gastrointestinal adverse events, hepatic adverse events, rash, QT prolongation, CYP2C9 and CYP3A4 inhibition; teratogenicity.

c Only for people with HIV.

d From CrAg+, asymptomatic individuals to obtunded patients requiring hospitalisation, including patients with renal impairment, anaemia, and/or neutropenia, and safe in pregnancy or whilst breastfeeding.

e BBB integrity is uncertain in patients with subclinical CM, but in patients with CM, increased permeability could allow non-CNS permeable compounds.

## References

[1] Pappas PG. Cryptococcal infections in non-HIV-infected patients. Trans Am Clin Clim Assoc 2013;124:61–79. PMC3715903 23874010

[2] Wang Y, Pawar S, Dutta O, Wang K, Rivera A, Xue C. Macrophage mediated immunomodulation during cryptococcus pulmonary infection. Front Cell Infect Microbiol 2022;12:859049. 10.3389/fcimb.2022.859049 PMC8987709 35402316

[3] Maziarz EK, Perfect JR. Cryptococcosis. Infect Dis Clin N Am 2016;30(1):179–206. 10.1016/j.idc.2015.10.006 PMC5808417 26897067

[4] Sabiiti W, May RC. Mechanisms of infection by the human fungal pathogen Cryptococcus neoformans. Future Microbiol 2012;7(11):1297–313. 10.2217/fmb.12.102 23075448

[5] Yoon HA, Felsen U, Wang T, Pirofski LA. Cryptococcus neoformans infection in human immunodeficiency virus (HIV)-infected and HIV-uninfected patients at an inner-city tertiary care hospital in the Bronx. Med Mycol 2020;58(4):434–43. 10.1093/mmy/myz082 31342058 PMC7261607

[6] Perfect JR, Casadevall A. Cryptococcosis. Infect Dis Clin N Am 2002;16(4):837–74. 10.1016/s0891-5520(02)00036-3. (v-vi). 12512184

[7] Jarvis JN, Lawrence DS, Meya DB, Kagimu E, Kasibante J, Mpoza E, Single-dose liposomal amphotericin B treatment for cryptococcal meningitis. N Engl J Med 2022;386(12):1109–20. 10.1056/NEJMoa2111904 35320642 PMC7612678

[8] Molloy SF, Kanyama C, Heyderman RS, Loyse A, Kouanfack C, Chanda D, Antifungal combinations for treatment of cryptococcal meningitis in Africa. N Engl J Med 2018;378(11):1004–17. 10.1056/NEJMoa1710922 29539274

[9] Mfinanga S, Kanyama C, Kouanfack C, Nyirenda S, Kivuyo SL, Boyer-Chammard T, Reduction in mortality from HIV-related CNS infections in routine care in Africa (DREAMM): a before-and-after, implementation study. Lancet HIV 2023;10(10):e663–73. 10.1016/S2352-3018(23)00182-0 37802567

[10] Ecevit IZ, Clancy CJ, Schmalfuss IM, Nguyen MH. The poor prognosis of central nervous system cryptococcosis among nonimmunosuppressed patients: a call for better disease recognition and evaluation of adjuncts to antifungal therapy. Clin Infect Dis 2006;42(10):1443–7. 10.1086/503570 16619158

[11] Zhou LH, Zhao HZ, Wang X, Wang RY, Jiang YK, Huang LP, Immune reconstitution inflammatory syndrome in non-HIV cryptococcal meningitis: cross-talk between pathogen and host. Mycoses 2021;64(11):1402–11. 10.1111/myc.13361 34390048 PMC9290805

[12] World Health Organization. WHO fungal priority pathogens list to guide research, development and public health action; 2022. 〈https://www.who.int/publications/i/item/9789240060241〉. [Accessed March 4, 2025].

[13] Chen SC-A, Meyer W, Sorrell TC. Cryptococcus gattii infections. Clin Microbiol Rev 2014;27(4):980–1024. 10.1128/CMR.00126-13 25278580 PMC4187630

[14] Yang DH, England MR, Salvator H, Anjum S, Park YD, Marr KA, Cryptococcus gattii species complex as an opportunistic pathogen: underlying medical conditions associated with the infection. mBio 2021;12(5):e0270821. 10.1128/mBio.02708-21 PMC8546560 34700378

[15] Rajasingham R, Govender NP, Jordan A, Loyse A, Shroufi A, Denning DW, The global burden of HIV-associated cryptococcal infection in adults in 2020: a modelling analysis. Lancet Infect Dis 2022;22(12):1748–55. 10.1016/S1473-3099(22)00499-6 36049486 PMC9701154

[16] Mirza SA, Phelan M, Rimland D, Graviss E, Hamill R, Brandt ME, The changing epidemiology of cryptococcosis: an update from population-based active surveillance in 2 large metropolitan areas, 1992–2000. Clin Infect Dis 2003;36(6):789–94. 10.1086/368091 12627365

[17] Pappas PG, Perfect JR, Cloud GA, Larsen RA, Pankey GA, Lancaster DJ, Cryptococcosis in human immunodeficiency virus-negative patients in the era of effective azole therapy. Clin Infect Dis 2001;33(5):690–9. 10.1086/322597 11477526

[18] Zhao Y, Ye L, Zhao F, Zhang L, Lu Z, Chu T, Cryptococcus neoformans, a global threat to human health. Infect Dis Poverty 2023;12(1):20. 10.1186/s40249-023-01073-4 36932414 PMC10020775

[19] Park BJ, Wannemuehler KA, Marston BJ, Govender N, Pappas PG, Chiller TM. Estimation of the current global burden of cryptococcal meningitis among persons living with HIV/AIDS. AIDS 2009;23(4):525–30. 10.1097/QAD.0b013e328322ffac 19182676

[20] Rajasingham R, Smith RM, Park BJ, Jarvis JN, Govender NP, Chiller TM, Global burden of disease of HIV-associated cryptococcal meningitis: an updated analysis. Lancet Infect Dis 2017;17(8):873–81. 10.1016/S1473-3099(17)30243-8 28483415 PMC5818156

[21] Denning DW. Global incidence and mortality of severe fungal disease. Lancet Infect Dis 2024;24(7):e428–38. 10.1016/S1473-3099(23)00692-8 38224705

[22] World Health Organization. Guidelines for diagnosing, preventing and managing cryptococcal disease among adults, adolescents and children living with HIV; 2022. 〈https://www.who.int/publications/i/item/9789240052178〉. [Accessed March 4, 2025]. 35797432

[23] Ngan NTT, Flower B, Day JN. Treatment of cryptococcal meningitis: how have we got here and where are we going? Drugs 2022;82(12):1237–49. 10.1007/s40265-022-01757-5 36112342 PMC9483520

[24] Cavassin FB, Baú-Carneiro JL, Vilas-Boas RR, Queiroz-Telles F. Sixty years of amphotericin B: an overview of the main antifungal agent used to treat invasive fungal infections. Infect Dis Ther 2021;10(1):115–47. 10.1007/s40121-020-00382-7 33523419 PMC7954977

[25] Gulati M, Bajad S, Singh S, Ferdous AJ, Singh M. Development of liposomal amphotericin B formulation. J Microencapsul 1998;15(2):137–51. 10.3109/02652049809006844 9532520

[26] Bausch Health Companies Inc. ANCOBON (flucytosine) capsules; 2022. 〈https://www.accessdata.fda.gov/drugsatfda_docs/label/2022/017001Orig1s034lbl.pdf〉. [Accessed April 2, 2025].

[27] Pfizer. Diflucan (fluconazole tablets) (fluconazole for oral suspension); 2024. 〈https://www.accessdata.fda.gov/drugsatfda_docs/label/2024/019949s072lbl.pdf〉. [Accessed April 2, 2025].

[28] Perfect JR, Dismukes WE, Dromer F, Goldman DL, Graybill JR, Hamill RJ, Clinical practice guidelines for the management of cryptococcal disease: 2010 update by the Infectious Diseases Society of America. Clin Infect Dis 2010;50(3):291–322. 10.1086/649858 20047480 PMC5826644

[29] Beardsley J, Wolbers M, Kibengo FM, Ggayi ABM, Kamali A, Cuc NTK, Adjunctive dexamethasone in HIV-associated cryptococcal meningitis. N Engl J Med 2016;374(6):542–54. 10.1056/NEJMoa1509024 26863355 PMC4778268

[30] Boulware DR, Meya DB, Muzoora C, Rolfes MA, Huppler Hullsiek K, Musubire A, Timing of antiretroviral therapy after diagnosis of cryptococcal meningitis. N Engl J Med 2014;370(26):2487–98. 10.1056/NEJMoa1312884 24963568 PMC4127879

[31] Bisson GP, Molefi M, Bellamy S, Thakur R, Steenhoff A, Tamuhla N, Early versus delayed antiretroviral therapy and cerebrospinal fluid fungal clearance in adults with HIV and cryptococcal meningitis. Clin Infect Dis 2013;56(8):1165–73. 10.1093/cid/cit019 23362285

[32] Makadzange AT, Ndhlovu CE, Takarinda K, Reid M, Kurangwa M, Gona P, Early versus delayed initiation of antiretroviral therapy for concurrent HIV infection and cryptococcal meningitis in sub-saharan Africa. Clin Infect Dis 2010;50(11):1532–8. 10.1086/652652 20415574

[33] Ingle SM, Miro JM, May MT, Cain LE, Schwimmer C, Zangerle R, Early antiretroviral therapy not associated with higher cryptococcal meningitis mortality in people with human immunodeficiency virus in high-income countries: an international collaborative cohort study. Clin Infect Dis 2023;77(1):64–73. 10.1093/cid/ciad122 36883578 PMC10320049

[34] Baddley JW, Forrest GN, AST Infectious Diseases Community of Practice. Cryptococcosis in solid organ transplantation - guidelines from the American Society of Transplantation Infectious Diseases Community of Practice. Clin Transpl 2019;33(9):e13543. 10.1111/ctr.13543 30900315

[35] Ssebambulidde K, Anjum SH, Hargarten JC, Chittiboina P, Shoham S, Seyedmousavi S, Treatment recommendations for non-HIV associated cryptococcal meningoencephalitis including management of post-infectious inflammatory response syndrome. Front Neurol 2022;13:994396. 10.3389/fneur.2022.994396 PMC9751747 36530631

[36] Izumikawa K, Kakeya H, Sakai F, Shibuya K, Sugita T, Takazono T, Executive summary of JSMM clinical practice guidelines for diagnosis and treatment of cryptococcosis 2019. Med Mycol J 2020;61(4):61–89. 10.3314/mmj.20.001 33250505

[37] Chang CC, Hall V, Cooper C, Grigoriadis G, Beardsley J, Sorrell TC, Consensus guidelines for the diagnosis and management of cryptococcosis and rare yeast infections in the haematology/oncology setting, 2021. Intern Med J 2021;51(7):118–42. 10.1111/imj.15590 34937137

[38] Chang CC, Harrison TS, Bicanic TA, Chayakulkeeree M, Sorrell TC, Warris A, Global guideline for the diagnosis and management of cryptococcosis: an initiative of the ECMM and ISHAM in cooperation with the ASM. Lancet Infect Dis 2024;24(8):e495–512. 10.1016/S1473-3099(23)00731-4 38346436 PMC11526416

[39] Cocco P, Ayaz-Shah A, Messenger MP, West RM, Shinkins B. Target product profiles for medical tests: a systematic review of current methods. BMC Med 2020;18(1):119. 10.1186/s12916-020-01582-1 32389127 PMC7212678

[40] Wake RM, Molloy SF, Jarvis JN, Harrison TS, Govender NP. Cryptococcal antigenemia in advanced human immunodeficiency virus disease: pathophysiology, epidemiology, and clinical implications. Clin Infect Dis 2023;76(4):764–70. 10.1093/cid/ciac675 35986670 PMC9938740

[41] Pullen MF, Hullsiek KH, Rhein J, Musubire AK, Tugume L, Nuwagira E, Cerebrospinal fluid early fungicidal activity as a surrogate endpoint for cryptococcal meningitis survival in clinical trials. Clin Infect Dis 2020;71(7):e45–9. 10.1093/cid/ciaa016 31912875 PMC7755087

[42] Montezuma-Rusca JM, Powers JH. Outcome assessments in clinical trials of cryptococcal meningitis: considerations on use of early fungicidal activity as a potential surrogate endpoint for all-cause mortality. Curr Treat Options Infect Dis 2014;6(3):326–36. 10.1007/s40506-014-0026-0 26306077 PMC4545574

[43] Tenforde MW, Mokomane M, Leeme T, Patel RKK, Lekwape N, Ramodimoosi C, Advanced human immunodeficiency virus disease in Botswana following successful antiretroviral therapy rollout: incidence of and temporal trends in cryptococcal meningitis. Clin Infect Dis 2017;65(5):779–86. 10.1093/cid/cix430 28505328 PMC5850554

[44] Anjum S, Dean O, Kosa P, Magone MT, King KA, Fitzgibbon E, Outcomes in previously healthy cryptococcal meningoencephalitis patients treated with pulse taper corticosteroids for post-infectious inflammatory syndrome. Clin Infect Dis 2021;73(9):e2789–98. 10.1093/cid/ciaa1901 33383587 PMC8563180

[45] World Health Organization. A framework for evaluating and publicly designating regulatory authorities as WHO Listed Authorities (WLA). 〈https://www.who.int/initiatives/who-listed-authority-reg-authorities〉. [Accessed March 4, 2025].

[46] Chang CC, Dorasamy AA, Gosnell BI, Elliott JH, Spelman T, Omarjee S, Clinical and mycological predictors of cryptococcosis-associated immune reconstitution inflammatory syndrome. AIDS 2013;27(13):2089–99. 10.1097/QAD.0b013e3283614a8d 23525034

[47] Wiesner DL, Boulware DR. Cryptococcus-related immune reconstitution inflammatory syndrome(IRIS): pathogenesis and its clinical implications. Curr Fungal Infect Rep 2011;5(4):252–61. 10.1007/s12281-011-0064-8 22389746 PMC3289516

[48] Shi ZW, Chen Y, Ogoke KM, Strickland AB, Shi M. Cryptococcal immune reconstitution inflammatory syndrome: from clinical studies to animal experiments. Microorganisms 2022;10(12):2419. 10.3390/microorganisms10122419 36557672 PMC9780901

[49] Roemer T, Krysan DJ. Antifungal drug development: challenges, unmet clinical needs, and new approaches. Cold Spring Harb Perspect Med 2014;4(5):a019703. 10.1101/cshperspect.a019703 PMC3996373 24789878

[50] Boulware DR, Atukunda M, Kagimu E, Musubire AK, Akampurira A, Tugume L, Oral lipid nanocrystal amphotericin B for cryptococcal meningitis: a randomized clinical trial. Clin Infect Dis 2023;77(12):1659–67. 10.1093/cid/ciad440 37606364 PMC10724459

[51] Lockhart SR, Fothergill AW, Iqbal N, Bolden CB, Grossman NT, Garvey EP, The investigational fungal Cyp51 inhibitor VT-1129 demonstrates potent in vitro activity against Cryptococcus neoformans and Cryptococcus gattii. Antimicrob Agents Chemother 2016;60(4):2528–31. 10.1128/AAC.02770-15 26787697 PMC4808209

[52] Wang L, Zhang M, Guo J, Guo W, Zhong N, Shen H, In vitro activities of the tetrazole VT-1161 compared with itraconazole and fluconazole against Cryptococcus and non-albicans Candida species. Mycologia 2021;113(5):918–25. 10.1080/00275514.2021.1913949 34132632

[53] Garvey EP, Sharp AD, Warn PA, Yates CM, Schotzinger RJ. The novel fungal CYP51 inhibitor VT-1598 is efficacious alone and in combination with liposomal amphotericin B in a murine model of cryptococcal meningitis. J Antimicrob Chemother 2018;73(10):2815–22. 10.1093/jac/dky242 29947783

[54] Murray A, Cass L, Ito K, Pagani N, Armstrong-James D, Dalal P, PC945, a novel inhaled antifungal agent, for the treatment of respiratory fungal infections. J Fungi 2020;6(4):373. 10.3390/jof6040373 PMC7765807 33348852

[55] Shaw KJ, Schell WA, Covel J, Duboc G, Giamberardino C, Kapoor M, In vitro and in vivo evaluation of APX001A/APX001 and other Gwt1 inhibitors against cryptococcus. Antimicrob Agents Chemother 2018;62(8):e00523–18. 10.1128/AAC.00523-18 PMC6105804 29891599

[56] Giamberardino CD, Schell WA, Tenor JL, Toffaletti DL, Palmucci JR, Marius C, Efficacy of APX2039 in a rabbit model of cryptococcal meningitis. mBio 2022;13(6):e0234722. 10.1128/mbio.02347-22 PMC9765414 36222509

[57] Wiederhold NP. Review of T-2307, an investigational agent that causes collapse of fungal mitochondrial membrane potential. J Fungi 2021;7(2):130. 10.3390/jof7020130 PMC7916847 33670132

[58] Koselny K, Green J, DiDone L, Halterman JP, Fothergill AW, Wiederhold NP, The celecoxib derivative AR-12 has broad-spectrum antifungal activity in vitro and improves the activity of fluconazole in a murine model of cryptococcosis. Antimicrob Agents Chemother 2016;60(12):7115–27. 10.1128/AAC.01061-16 27645246 PMC5118990

[59] Vahedi-Shahandashti R, Lass-Flörl C. In vitro activity of SF001: a next-generation polyene versus amphotericin B. Antimicrob Agents Chemother 2025;69(6):e00322–25. 10.1128/aac.00322-25 PMC12135503 40261080

[60] Li L, Wu H, Zhu S, Ji Z, Chi X, Xie F, Discovery of novel 7-hydroxy-5-oxo-4,5-dihydrothieno[3,2-b]pyridine-6-carboxamide derivatives with potent and selective antifungal activity against Cryptococcus species. J Med Chem 2022;65(16):11257–69. 10.1021/acs.jmedchem.2c00794 35922963

[61] Kryštůfek R, Šácha P, Starková J, Brynda J, Hradilek M, Tloušt’ová E, Reemerging aspartic protease targets: examining Cryptococcus neoformans major aspartyl peptidase 1 as a target for antifungal drug discovery. J Med Chem 2021;64(10):6706–19. 10.1021/acs.jmedchem.0c02177 34006103 PMC8165695

[62] Donlin MJ, Lane TR, Riabova O, Lepioshkin A, Xu E, Lin J, Discovery of 5-nitro-6-thiocyanatopyrimidines as inhibitors of Cryptococcus neoformans and Cryptococcus gattii. ACS Med Chem Lett 2021;12(5):774–81. 10.1021/acsmedchemlett.1c00038 34055225 PMC8155264

[63] Iyer KR, Li SC, Revie NM, Lou JW, Duncan D, Fallah S, Identification of triazenyl indoles as inhibitors of fungal fatty acid biosynthesis with broad-spectrum activity. Cell Chem Biol 2023;30(7):795–810.e8. 10.1016/j.chembiol.2023.06.005 37369212 PMC11016341

[64] Puumala E, Fallah S, Robbins N, Cowen LE. Advancements and challenges in antifungal therapeutic development. Clin Microbiol Rev 2024;37(1):e0014223. 10.1128/cmr.00142-23 PMC10938895 38294218

[65] Scannell JW, Bosley J, Hickman JA, Dawson GR, Truebel H, Ferreira GS, Predictive validity in drug discovery: what it is, why it matters and how to improve it. Nat Rev Drug Discov 2022;21(12):915–31. 10.1038/s41573-022-00552-x 36195754

[66] De Rycker M, Baragaña B, Duce SL, Gilbert IH. Challenges and recent progress in drug discovery for tropical diseases. Nature 2018;559(7715):498–506. 10.1038/s41586-018-0327-4 30046073 PMC6129172

[67] Robertson EJ, Najjuka G, Rolfes MA, Akampurira A, Jain N, Anantharanjit J, Cryptococcus neoformans ex vivo capsule size is associated with intracranial pressure and host immune response in HIV-associated cryptococcal meningitis. J Infect Dis 2014;209(1):74–82. 10.1093/infdis/jit435 23945372 PMC3864387

[68] Kovanda LL, Giamberardino C, McEntee L, Toffaletti DL, Franke KS, Bartuska A, Pharmacodynamics of isavuconazole in a rabbit model of cryptococcal meningoencephalitis. Antimicrob Agents Chemother 2019;63(9):e00546–19. 10.1128/AAC.00546-19 PMC6709487 31209006

[69] Lestner J, McEntee L, Johnson A, Livermore J, Whalley S, Schwartz J, Experimental models of short courses of liposomal amphotericin B for induction therapy for cryptococcal meningitis. Antimicrob Agents Chemother 2017;61(6):e00090–17. 10.1128/AAC.00090-17 PMC5444125 28320715

[70] Sionov E, Chang YC, Garraffo HM, Kwon-Chung KJ. Heteroresistance to fluconazole in Cryptococcus neoformans is intrinsic and associated with virulence. Antimicrob Agents Chemother 2009;53(7):2804–15. 10.1128/AAC.00295-09 19414582 PMC2704677

[71] Livermore J, Howard SJ, Sharp AD, Goodwin J, Gregson L, Felton T, Efficacy of an abbreviated induction regimen of amphotericin B deoxycholate for cryptococcal meningoencephalitis: 3 days of therapy is equivalent to 14 days. mBio 2014;5(1):e00725–13. 10.1128/mBio.00725-13 24473125 PMC3903272

[72] Giamberardino CD, Tenor JL, Toffaletti DL, Palmucci JR, Schell W, Boua J-VK, Pharmacodynamics of ATI-2307 in a rabbit model of cryptococcal meningoencephalitis. Antimicrob Agents Chemother 2023;67(10):e0081823. 10.1128/aac.00818-23 PMC10583688 37728934

[73] Pang PD, Ahmed SM, Nishiga M, Stockbridge NL, Wu JC. Tackling the challenges of new approach methods for predicting drug effects from model systems. Nat Rev Drug Discov 2024;23(8):565–6. 10.1038/d41573-024-00081-9 38750208 PMC11482555

[74] Roosen L, Maes D, Musetta L, Himmelreich U. Preclinical models for cryptococcosis of the CNS and their characterization using in vivo imaging techniques. J Fungi 2024;10(2):146. 10.3390/jof10020146 PMC10890286 38392818

[75] Zaragoza O, García-Rodas R, Nosanchuk JD, Cuenca-Estrella M, Rodríguez-Tudela JL, Casadevall A. Fungal cell gigantism during mammalian infection. PLoS Pathog 2010;6(6):e1000945. 10.1371/journal.ppat.1000945 PMC2887474 20585557

[76] Chastain DB, Rao A, Yaseyyedi A, Henao-Martínez AF, Borges T, Franco-Paredes C. Cerebral cryptococcomas: a systematic scoping review of available evidence to facilitate diagnosis and treatment. Pathogens 2022;11(2):205. 10.3390/pathogens11020205 35215148 PMC8879191

[77] Charlier C, Chrétien F, Baudrimont M, Mordelet E, Lortholary O, Dromer F. Capsule structure changes associated with Cryptococcus neoformans crossing of the blood-brain barrier. Am J Pathol 2005;166(2):421–32. 10.1016/S0002-9440(10)62265-1 15681826 PMC1602336

[78] European Medicines Agency. Guideline on the clinical evaluation of antifungal agents for the treatment and prophylaxis of invasive fungal disease; 2010. 〈https://www.ema.europa.eu/en/documents/scientific-guideline/guideline-clinical-evaluation-antifungal-agents-treatment-and-prophylaxis-invasive-fungal-disease_en.pdf〉. [Accessed March 4, 2025].

[79] Pallmann P, Bedding AW, Choodari-Oskooei B, Dimairo M, Flight L, Hampson LV, Adaptive designs in clinical trials: why use them, and how to run and report them. BMC Med 2018;16(1):29. 10.1186/s12916-018-1017-7 29490655 PMC5830330

[80] Marr KA, Sun Y, Spec A, Lu N, Panackal A, Bennett J, A multicenter, longitudinal cohort study of cryptococcosis in human immunodeficiency virus-negative people in the United States. Clin Infect Dis 2020;70(2):252–61. 10.1093/cid/ciz193 30855688 PMC6938979

[81] Pirofski LA, Casadevall A. Immune-mediated damage completes the parabola: Cryptococcus neoformans pathogenesis can reflect the outcome of a weak or strong immune response. mBio 2017;8(6):e02063–17. 10.1128/mBio.02063-17 PMC5727418 29233901

[82] de Oliveira L, Melhem MSC, Buccheri R, Chagas OJ, Vidal JE, Diaz-Quijano FA. Early clinical and microbiological predictors of outcome in hospitalized patients with cryptococcal meningitis. BMC Infect Dis 2022;22(1):138. 10.1186/s12879-022-07118-7 35139801 PMC8830130

[83] Loyse A, Dromer F, Day J, Lortholary O, Harrison TS. Flucytosine and cryptococcosis: time to urgently address the worldwide accessibility of a 50-year-old antifungal. J Antimicrob Chemother 2013;68(11):2435–44. 10.1093/jac/dkt221 23788479 PMC3797641

[84] Bermas A, Geddes-McAlister J. Combatting the evolution of antifungal resistance in Cryptococcus neoformans. Mol Microbiol 2020;114(5):721–34. 10.1111/mmi.14565 32697029

[85] Bongomin F, Oladele RO, Gago S, Moore CB, Richardson MD. A systematic review of fluconazole resistance in clinical isolates of Cryptococcus species. Mycoses 2018;61(5):290–7. 10.1111/myc.12747 29377368

[86] Singh N, Huprikar S, Burdette SD, Morris MI, Blair JE, Wheat LJ, Donor-derived fungal infections in organ transplant recipients: guidelines of the American Society of Transplantation, infectious diseases community of practice. Am J Transpl 2012;12(9):2414–28. 10.1111/j.1600-6143.2012.04100.x 22694672

[87] Zafar H, Altamirano S, Ballou ER, Nielsen K. A titanic drug resistance threat in Cryptococcus neoformans. Curr Opin Microbiol 2019;52:158–64. 10.1016/j.mib.2019.11.001 31765991 PMC6941473

[88] Stone NR, Rhodes J, Fisher MC, Mfinanga S, Kivuyo S, Rugemalila J, Dynamic ploidy changes drive fluconazole resistance in human cryptococcal meningitis. J Clin Investig 2019;129(3):999–1014. 10.1172/JCI124516 30688656 PMC6391087

[89] Sionov E, Lee H, Chang YC, Kwon-Chung KJ. Cryptococcus neoformans overcomes stress of azole drugs by formation of disomy in specific multiple chromosomes. PLoS Pathog 2010;6(4):e1000848. 10.1371/journal.ppat.1000848 PMC2848560 20368972

[90] Iyer KR, Revie NM, Fu C, Robbins N, Cowen LE. Treatment strategies for cryptococcal infection: challenges, advances and future outlook. Nat Rev Microbiol 2021;19(7):454–66. 10.1038/s41579-021-00511-0 33558691 PMC7868659

